# Neck Circumference Predicts the Occurrence and Remission of Metabolic Associated Fatty Liver Disease: A Longitudinal Study of Community-Dwelling Population

**DOI:** 10.1159/000526075

**Published:** 2022-07-20

**Authors:** Weijie Cao, Yiting Xu, Yun Shen, Yufei Wang, Xiaojing Ma, Yuqian Bao

**Affiliations:** Department of Endocrinology and Metabolism, Shanghai Diabetes Institute, Shanghai Clinical Center for Diabetes, Shanghai Key Clinical Center for Metabolic Disease, Shanghai Key Laboratory of Diabetes Mellitus, Shanghai Jiao Tong University Affiliated Sixth People's Hospital, Shanghai, China

**Keywords:** Metabolism associated fatty liver disease, Neck circumference, Obesity, Longitudinal study

## Abstract

**Aim:**

Neck circumference (NC), a proxy for upper-body subcutaneous fat, is closely related to metabolic dysfunction, independent of other obesity indices. The purpose of this study was to explore the relationship between NC and the incidence and remission of metabolic associated fatty liver disease (MAFLD), a novel concept proposed by an international consensus panel in 2020 through a community-based longitudinal cohort.

**Methods:**

This study included 1,549 community participants and was conducted from 2013 to 2016. MAFLD was diagnosed using the International Expert Consensus (2020) criteria. All participants underwent NC measurement and biochemical measurements. Elevated NC was defined as NC ≥38.5 cm in men and NC ≥34.5 cm in women.

**Results:**

A total of 1,549 subjects (638 men and 911 women), with an average age of 59.6 ± 7.3 years, were included. During a mean follow-up of 2.1 years, MAFLD occurred in 146 of the 870 participants without baseline MAFLD and was resolved in 225 of the 679 participants with baseline MAFLD. After adjusting for confounding factors such as age, sex, body mass index, waist circumference, fasting plasma glucose, and liver enzyme levels, multivariable logistic regression showed that higher NC at baseline was positively correlated with MAFLD occurrence (OR 1.96, 95% confidence interval: 1.21–3.31; *p* = 0.003) and negatively correlated with MAFLD remission (OR 0.57, 95% confidence interval: 0.40–0.80; *p* < 0.001).

**Conclusion:**

A higher NC is associated with an increased risk of MAFLD occurrence and a reduced probability of MAFLD remission, making NC measurement a potential predictor in MAFLD management.

## Introduction

Obesity has become a global public health concern, and China has the largest number of affected people worldwide, with more than 50% of adults being obese or overweight [1, 2]. With a deeper understanding of obesity, fat distribution was found to be a stronger determinant of metabolic health than increased fat mass itself [3]. Neck circumference (NC), a convenient anthropometric index, can reflect fat deposition in the upper body [4, 5]. Previous longitudinal studies indicated that elevated NC was significantly associated with an increased risk of metabolic dysfunction such as type 2 diabetes, hyperuricemia, and insulin resistance [6, 7, 8].

In 2020, an international panel of experts proposed “metabolic associated fatty liver disease” (MAFLD) as a novel concept, regardless of alcohol intake and other concomitant liver diseases, to highlight the key role of metabolic dysfunction in the underlying causes, manifestations, progression, and outcomes of fatty liver diseases [9]. Fatty liver includes a wide range of histopathological features, from hepatic steatosis to steatohepatitis, hepatocyte injury, and hepatic fibrosis [10]. The early stage of fatty liver (hepatic steatosis) is reversible and benign; however, once it progresses to steatohepatitis, the risk associated with liver cancer and death is conspicuously increased [11]. Therefore, identifying those at high risk of MAFLD in the general population and the MAFLD patients who are less likely to regress to normal is of prime importance in the prevention and treatment of MAFLD.

Previous cross-sectional studies concluded that NC might be a simple index of fatty liver [12, 13, 14], but whether neck fat accumulation could predict the occurrence and remission of MAFLD remains unknown. This study aimed to explore the relationship between NC and the occurrence and remission of MAFLD through a community-based longitudinal cohort.

## Materials and Methods

### Study Population

**Fig. 1:**
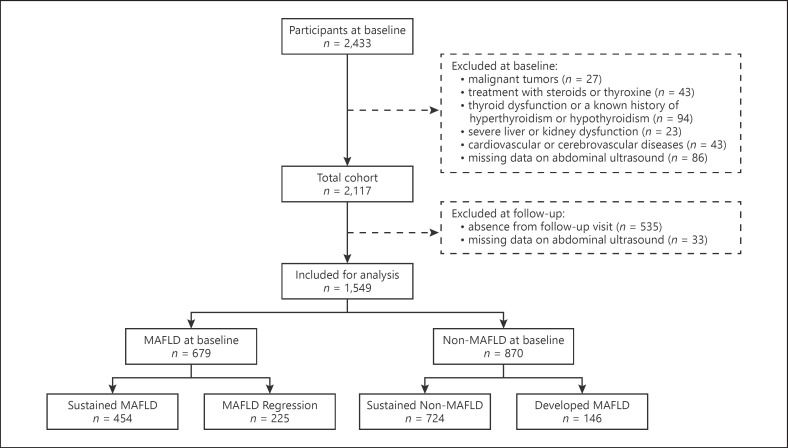
Flow chart of study population.

We enrolled participants from communities in Shanghai between 2013 and 2014. The collected data were derived from standardized questionnaires and included information on current and previous illnesses, medications, physical examinations, and biochemical measurements. We then conducted a follow-up of participants from 2015 to 2016. Participants with a known history of cardiovascular or cerebrovascular diseases, malignant tumors, thyroid dysfunction or a history of hyperthyroidism/hypothyroidism, treatment with steroids or thyroxine, severe liver and kidney dysfunction, and absence of liver ultrasound were excluded. A total of 2,177 eligible participants with complete data were recruited between 2013 and 2014. The participants were followed up for 1.1–2.9 years with an average of 2.1 ± 0.2 years from 2015 to 2016, and we excluded those with absence of follow-up visit due to migration or missing data of abdominal ultrasound (Fig. 1). This study was approved by the Ethics Committee of the Sixth People's Hospital Affiliated to Shanghai Jiao Tong University, and all participants provided written informed consent before participation.

### Anthropometric and Biochemical Measurements

All anthropometric indices and biochemical measurements were collected at the baseline and during follow-up. Height, weight, waist circumference (WC), and resting blood pressure were measured using standardized methods by trained researchers [15]. Body mass index (BMI) was calculated as weight (kg)/height^2^ (m^2^) The measurement of NC was performed by trained researchers when the participants were standing with their head in a horizontal plane. The upper edge of the tape was positioned below the thyroid cartilage protrusion and perpendicular to the long axis of the neck (avoiding compression of the skin), and then the circumference of the neck was recorded. The minimum circumference was recorded to the nearest 0.1 cm. Elevated NC was defined according to our previous study: NC ≥38.5 cm in men and NC ≥34.5 cm in women [15]. Elevated WC was defined by the criteria for central obesity: WC ≥90 cm in men and WC ≥85 cm in women [16].

All participants had been instructed to fast for 10 h before the examination. Venous blood samples were collected by professional nurses. Biochemical variables, namely fasting plasma glucose (FPG), fasting insulin (FINS), total cholesterol (TC), triglycerides (TG), high-density lipoprotein cholesterol (HDL-c), low-density lipoprotein cholesterol (LDL-c), C-reactive protein (CRP), alanine aminotransferase (ALT), alanine aminotransferase (AST), alkaline phosphatase (ALP), glutamyl transferase (GGT), creatinine (CR), and glycated hemoglobin (HbA_1c_) levels, were determined using fasting blood samples after an overnight fast using standard methods [17]^.^ Thereafter, participants without diabetes underwent a 75-g oral glucose tolerance test, and those with diabetes took the 100-g steamed bread meal test; the 2-h blood glucose (2hPG) level was subsequently measured. The homeostasis model assessment of insulin resistance (HOMA-IR) was as follows: HOMA-IR = FINS (µU/L) × FPG (mmol/L)/22.5 [18].

### Diagnostic Criteria of MAFLD

All participants underwent abdominal ultrasonographic examination using a Voluson 730 Expert B-mode ultrasonographic device (5.0-MHz transducer, GE Healthcare, Waukesha, WI, USA) by the same trained sonographer at baseline and follow-up, who was blinded to the study design and clinical details of the participants. Fatty liver was diagnosed through ultrasonography and the presence of at least two of the following four findings: (1) diffusely increased echogenicity of the liver relative to the kidney or spleen; (2) ultrasound beam attenuation with decreased vessel signal; (3) poor visualization of intrahepatic structures; and (4) slightly enlarged liver with a blunt margin. In this study, MAFLD was diagnosed according to the International Expert Consensus statement on MAFLD in 2020 [9]and combined with the presence of any one of the following three conditions: overweight/obesity (BMI ≥23 kg/m^2^), type 2 diabetes, or clues of metabolic dysregulation. Metabolic dysfunction was defined as the presence of at least two of the following metabolic risk factors: (1) WC ≥90 cm in men and ≥80 cm in women; (2) blood pressure ≥130/85 mm Hg or antihypertensive therapy; (3) TG ≥1.7 mmol/L or lipid-lowering therapy; (4) HDL-c<1 mmol/L for men and <1.3 mmol/L for women or drug therapy; (5) prediabetes, i.e., an FPG of 5.6–6.9 mmol/L, a 2hPG of 7.8–11.0 mmol/L, or an HbA_1c_ of 5.7–6.4%; (6) HOMA-IR ≥2.5; and (7) plasma CRP level >2 mg/L.

Hypertension was defined as systolic BP ≥140 mm Hg, diastolic BP ≥90 mm Hg, and/or use of antihypertensive medications [19]. Dyslipidemia was defined as at least one of the following criteria: (1) TC ＞5.2 mmol/L (200 mg/dL); (2) TG ≥1.7 mmol/L (150 mg/dL); (3) LDL-c ≥3.4 mmol/L (130 mg/dL); (4) HDL-c <1.0 mmol/L (40 mg/dL); and (5) use of lipid-lowering medications [20]. Individuals with diabetes were diagnosed according to the 2010 American Diabetes Association criteria [21].

### Statistical Analyses

Statistical analysis was performed using SPSS 20.0 (IBM SPSS Inc., Chicago, IL, USA), and a two-tailed *p* < 0.05 was considered statistically significant. Normally distributed data, skewed data, and categorical variables are shown as the mean ± standard deviation, median (interquartile range), and number (percentages), respectively. Student's *t* test was used to compare two groups with normal distribution, whereas the Wilcoxon rank sum test was used to compare two groups with skewed distribution. Multivariable logistic regression was used to analyze the relationship between NC and MAFLD occurrence and remission. NC was treated as a categorical variable (higher or lower). Subgroup analyses according to sex (men vs. women), age (<65 vs. ≥65 years), BMI (<24 vs. ≥24 kg/m^2^), and WC (<90 cm in men and <85 cm in women vs. ≥90 cm in men and ≥85 cm in women) were further explored to test result consistency among different subgroups.

## Results

### Clinical Characteristics of Study Participants

A total of 1,549 (638 men and 911 women) subjects aged between 30 and 80 years, with an average age of 59.6 ± 7.3 years, with complete data were included. The prevalence of MAFLD at baseline was 43.83%, and the prevalence of hypertension, diabetes, and dyslipidemia at baseline was 48.93%, 19.74%, and 32.92%, respectively. The participants were followed up for 1.1–2.9 years, with an average of 2.1 ± 0.2 years. The baseline characteristics of the participants are presented in Table 1.

Among 870 participants without MAFLD at baseline, 146 (16.78%) developed MAFLD at follow-up. Participants who developed MAFLD had significantly higher levels of BMI, WC, NC, SBP, DBP, FPG, 2hPG, FINS, HOMA-IR, HbA_1c_, ALT, GGT, TG, and Cr at baseline than those who remained normal (all *p* < 0.05). Additionally, the HDL-c level of those who developed MAFLD was significantly lower at baseline than those who remained normal (*p* < 0.01).

Of the 679 subjects with MAFLD at baseline, 225 (33.14%) showed MAFLD remission during follow-up. Participants remaining with MAFLD had significantly higher levels of BMI, WC, NC, SBP, DBP, FPG, 2hPG, FINS, HOMA-IR, HbA_1c_, ALT, GGT, TG, and Cr at baseline than those with MAFLD remission (all *p* < 0.05). Furthermore, the HDL-c level of those remaining with MAFLD was significantly lower at baseline than those with MAFLD remission at follow-up (*p* < 0.01).

### Association between Baseline NC and MAFLD Occurrence at Follow-Up

**Fig. 2:**
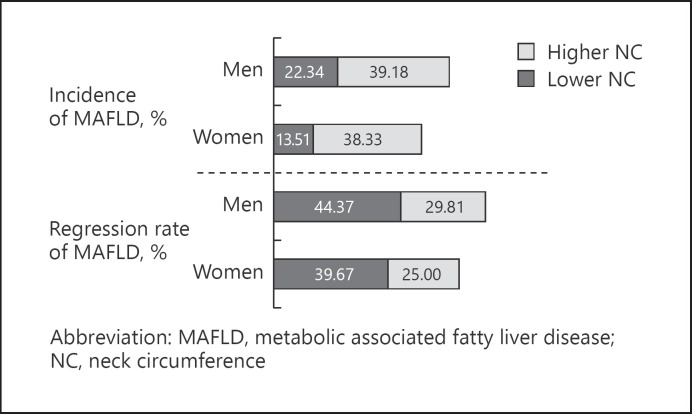
Incidence or remission rate of MAFLD in different NC groups.

The study subjects were further divided into two groups according to NC. The baseline lower NC group was defined as: NC <38.5 cm in men and NC <34.5 cm in women. The baseline higher NC group was defined as: NC ≥38.5 cm in men and NC ≥34.5 cm in women. The incidence of MAFLD in the higher NC group was significantly higher compared to that of the lower NC group in both men and women (39.18% vs. 22.34% in men; 38.33% vs. 13.51% in women, both *p* < 0.01) (Fig. 2).

**Fig. 3:**
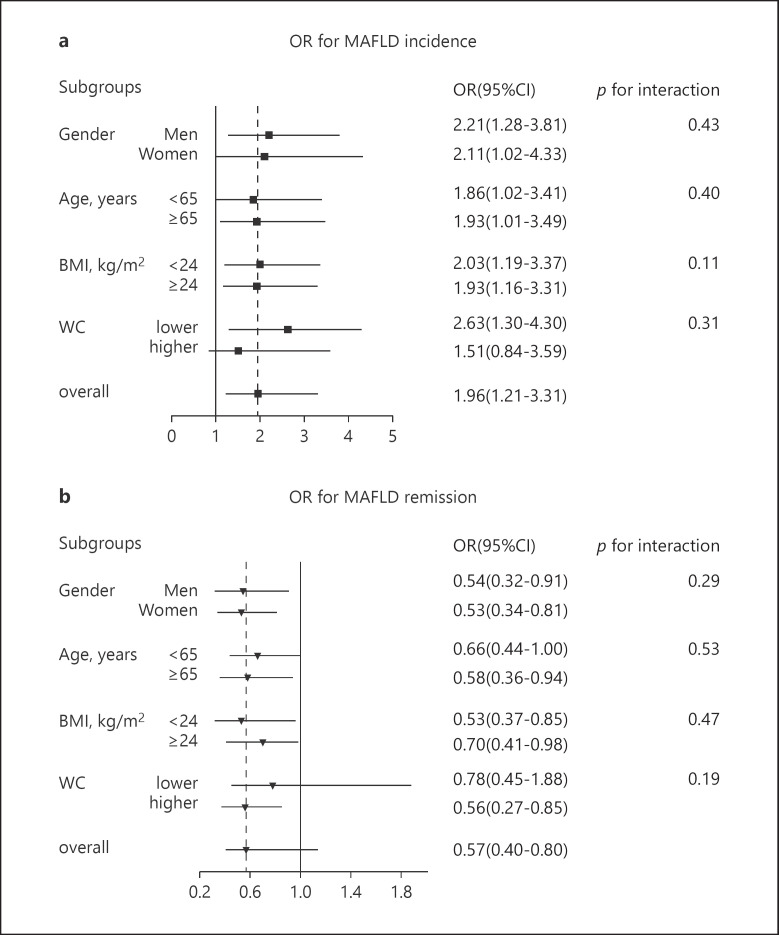
Subgroup analyses by gender (men vs. women), age (<65 vs. ≥65 years), BMI (<24 vs. ≥24 kg/m^2^), and WC (<90 cm in men and 85 cm in women vs. ≥90 cm in men and 85 cm in women) using multivariable logistic regression. The model was adjusted for age, gender, BMI category, WC category, HOMA-IR, CRP, fasting plasma glucose, post-loading plasma glucose lipid profiles, liver enzymes, hypertension, diabetes, and dyslipidemia, except the stratified variable. The data are shown as the adjusted odds ratio (95% confidence interval) in higher NC levels compared to lower NC levels for the risk of MAFLD incidence (**a**) and for the probability of MAFLD remission (**b**).

To further explore the association between NC and the occurrence of MAFLD, we performed a logistic regression analysis, in which the occurrence of MAFLD was designated as the dependent variable. After adjusting for age, sex, BMI, WC, HOMA-IR, CRP, FPG, 2hPG, ALT, AST, GGT, hypertension, diabetes, and dyslipidemia, higher NC at baseline was a positive factor for the occurrence of MAFLD (OR 1.96, 95% CI: 1.21–3.31, *p* < 0.01). We then analyzed whether NC was associated with MAFLD in the different subgroups. Subgroup analyses were stratified by gender (men vs. women), age (<65 vs. ≥65 years), BMI (<24 kg/m^2^ vs. ≥24 kg/m^2^), and WC (<90 cm in men and 85 cm in women vs. ≥90 cm in men and 85 cm in women) using multivariable logistic regression. Except for the higher WC groups, higher NC at baseline was positively correlated with the occurrence of MAFLD and a positive correlation was more significant in the lower BMI and lower WC groups (Fig. 3a).

### Association between Baseline NC and MAFLD Remission at Follow-Up

The remission rate of MAFLD in the higher NC group was diminished compared to that in the lower NC group (29.81% vs. 44.37% in men; 25.00% vs. 39.67% in women; both *p* < 0.01) (Fig. 2). To further explore the association between NC and MAFLD remission, we also performed a logistic regression analysis, in which MAFLD remission was set as the dependent variable. After adjusting for age, sex, BMI, WC, HOMA-IR, CRP, FPG, 2hPG, ALT, AST, GGT, hypertension, diabetes, and dyslipidemia, higher NC at baseline was a negative factor for the remission of MAFLD (OR 0.57, 95% CI: 0.40–0.80, *p* < 0.01). We then analyzed whether NC was associated with MAFLD remission in the different subgroups. Except for the lower WC groups, higher NC at baseline was negatively correlated with the possibility of MAFLD remission in all groups, and the inverse correlation was more obvious in the lower BMI and higher WC groups (Fig. 3b).

## Discussion

Our study showed that the risk of MAFLD occurrence increased by 96%, and the possibility of MAFLD remission decreased by 43% in the higher NC community population after an average follow-up of 2.1 years. This indicated the importance of neck fat accumulation in predicting the occurrence and remission of MAFLD.

A new positive definition for MAFLD was proposed by an international expert consensus in 2020. The expanded criteria of inclusion, rather than exclusion, are expected to provide a more comprehensive overview of the widespread hepatic steatosis disease and emphasize the contribution of metabolic diseases to the presence and progression of hepatic steatosis. The International Expert Consensus hopes to identify participants at the early stages of metabolic dysfunction and those at a higher risk of disease progression through this definition. After the concept of MAFLD was proposed, a study involving 765 Japanese revealed that using the definition of MAFLD rather than that of non-alcoholic fatty liver disease (NAFLD) led to better identification of liver stiffness evaluated by non-invasive methods including Fibro-Scan and FIB-4 index [22]. In addition, a study enrolled more than 8,000 Chinese individuals with an average follow-up of 4 years and assessed subclinical atherosclerosis by ultrasonic determination of carotid intima-media thickness and brachial-ankle pulse wave velocity. The results suggested that individuals with MAFLD remission had a much lower risk of subclinical atherosclerosis than those without MAFLD remission [23].

As a simple anthropometric index for assessing upper-body fat accumulation, the measurement of NC is simple and minimally affected by breathing and diet, with an explicit anatomic landmark, high repeatability, and low variability. Increasing evidence has implied a strong association between NC and metabolic dysfunction such as type 2 diabetes, insulin resistance, and hyperuricemia. A cross-sectional study of 2,761 subjects in China showed that NC was significantly positively correlated with fasting insulin levels and HOMA-IR in men and women [24]. Another study included 4,383 people without hyperuricemia at baseline and demonstrated that after a 3-year follow-up, there was a positive correlation between NC at baseline and serum uric acid at follow-up, and higher NC increased the risk of hyperuricemia in women by 48% [6].

The NC cutoff points used in the current study were obtained from our previous study [15]. We assessed central obesity using the precise standard-visceral fat area, which was measured with magnetic resonance imaging, and proposed that the optimal NC cutoff points for metabolic dysfunction were 38.5 cm for men and 34.5 cm for women. We found that the higher NC group, compared to the lower NC group, had an increased risk of MAFLD occurrence and a reduced possibility of MAFLD remission. A recent study including 1,354 postmenopausal women in China with normal BMI (18.5–25.0 kg/m^2^) demonstrated that higher NC was associated with an increased risk of NAFLD after an average follow-up of 3.1 years [25]. In this current study, we conducted a stratified analysis according to sex, age, BMI, and WC. After stratification by sex, age, BMI and WC, the positive correlation between high NC and MAFLD occurrence became more obvious in the lower BMI and lower WC groups, and the inverse association between higher NC and MAFLD remission was more obvious in the lower BMI and higher WC groups. Interestingly, we did not observe that NC was associated with the risk of MAFLD in the higher WC group, while the lower WC group showed NC was not associated with the possibility of MAFLD remission. This indicated that NC may play diverse roles in predicting the occurrence and remission of MAFLD in different WC populations, which requires further study. Previous cross-sectional studies have found that NC is associated with fatty liver [12, 13, 14]. Our longitudinal study could verify the results and provide valuable clues for future research.

It is generally acknowledged that WC and BMI are the most popular indicators in evaluating obesity [2]. NC is another anthropometric index to evaluate obesity proposed in recent years [5, 6, 7]. Accumulating studied explored relationship between NC and metabolic indicators or fat distribution. A Spanish study including 119 young adults showed that the association between NC and indicators of body composition was, however, weaker than that observed by BMI and WC [26]. Meta analysis found no association between NC and metabolic syndrome while there was a positive association between NC and WC, BMI, TG, TC, SBP, and FPG [27, 28]. Despite the debate on NC, NC has its own advantages such as a simple method with explicit anatomic landmark to measure, being minimally affected by breathing and diet, a high repeatability, and low variability. Future studies may focus on larger samples and longer follow-up to detect the reliability of NC.

The underlying mechanism behind NC and MAFLD remains unclear. NC is a great indicator of ectopic fat distribution as an alternative measurement of upper-body subcutaneous fat. Subcutaneous fat in the upper body accounts for a greater proportion of systemic free fatty acids released and is more lipolytically active than lower body adipose tissue [29]. Elevated NC indicates excessive accumulation of subcutaneous fat in the neck, which contributes to a greater flux of free fatty acids released into circulation. Subsequently, elevated free fatty acids contribute to increased synthesis and ectopic deposition of triglycerides, insulin resistance, and inflammation [30]. Additionally, there is a complex crosstalk between fat homeostasis and the liver's regional immune system. Proinflammatory macrophages in neck adipose tissue decrease hepatocyte responsiveness to insulin by impairing insulin-mediated phosphorylation of insulin receptor substrate 1 (IRS1) and IRS1-associated PI3K in hepatocytes and produce high levels of neutrophil chemotactic proteins, thus contributing to increased hepatic neutrophil and macrophage infiltration and worsening liver damage [31].

This study had limitations. First, similar to most epidemiological studies, there may be selection bias. Our study is a single-center study, which only included the community population in Shanghai. Further studies based on other regions and ethnic groups are needed to verify our results. Second, considering that the diagnosis of fatty liver was based on ultrasound imaging, it was difficult to evaluate the stage of fatty liver.

## Conclusion

In conclusion, subjects with higher NC had an increased risk of MAFLD occurrence and a reduced possibility of MAFLD remission, indicating that NC was a potential index in MAFLD. NC measurement can help us better predict the occurrence and remission of MAFLD.

## Statement of Ethics

The study was reviewed and approved by the Ethics Committee of Shanghai Jiao Tong University Affiliated Sixth People's Hospital, approval number 2019-067. All procedures were performed in accordance with the 1964 Declaration of Helsinki and its later amendments or comparable ethical standards. The study methods and potential risks were fully explained to all participants, and each participant provided a written informed consent prior to enrollment.

## Conflict of Interest Statement

The authors declare that they have no conflict of interest.

## Funding Sources

This work was funded by the Shanghai Municipal Science and Technology Commission Medical Guide Project (19411964300).

## Authors Contributions

Xiaojing Ma and Yuqian Bao conceived the work. Weijie Cao and Yiting Xu performed the statistical analyses. Yiting Xu, Yun Shen, Yufei Wang, and Xiaojing Ma contributed to data collection. Weijie Cao and Yiting Xu contributed to drafting the article. Xiaojing and Yuqian Bao revised the manuscript.

## Data Availability Statement

The data used to support the findings of this study are available from the corresponding author upon request.

## Figures and Tables

**Table 1:** Baseline characteristics of study population according to development or regression of MAFLD at follow-up

Characteristics	Incident MAFLD (*n* = 870)		MAFLD regression (*n* = 679)	
	yes (*n* = 146)	no (*n* = 724)	yes (*n* = 225)	no (*n* = 454)
Men/women	83/63	291/433	95/130	169/285
Age, years	58.6 (54.1–63.1)	58.4 (53.5–63.2)	60.6 (55.6–64.8)	58.8 (53.8–63.5)
BMI, kg/m^2^	24.0 (22.5–25.8)	22.4 (20.7–24.2)**	25.0 (23.7–26.7)	26.0 (24.3–28.2)**
NC, cm				
Men	37.9±1.8	37.1±2.1**	38.6±2.0	39.6±2.4**
Women	33.8±2.1	32.7±1.7**	34.3±1.7	35.0±1.9**
WC, cm				
Men	88.0±6.6	83.3±7.6**	90.0±7.2	94.3±8.5**
Women	82.9±7.9	76.7±7.1**	85.2±6.4	88.0±7.4**
SBP, mm Hg	134±18	129±18**	135±17	137±19*
DBP, mm Hg	81±10	78±10**	80±10	82±11 *
FPG, mmol/L	5.4 (5.0–6.0)	5.1 (4.8–5.5)*	5.3 (4.9–5.8)	5.5 (5.1–6.3)**
2hPG, mmol/L	7.4 (6.0–9.2)	6.7 (5.6–8.4)**	7.8 (6.2–10.1)	8.1 (6.7–10.9)**
FINS, μU/mL	8.5 (6.9–12.3)	6.2 (4.5–8.2)**	9.0 (6.4–11.9)	11.9 (9.1–16.3)**
HbA_1c_, %	5.7 (5.4–6.0)	5.6 (5.3–5.8)*	5.7 (5.4–6.0)	5.8 (5.5–6.2)**
HOMA-IR	2.1 (1.6–3.0)	1.4 (1.0–2.0)**	2.2 (1.5–3.1)	3.2 (2.2–4.3)**
ALT, U/L	17 (13–23)	15 (12–20)**	18 (14–23)	22 (16–32)**
AST, U/L	20 (17–23)	20 (17–24)	21 (18–25)	22 (19–27)*
GGT, U/L	25 (19–35)	19 (15–27)**	25 (17–37)**	30 (22–45)**
TC, mmol/L	5.0±0.9	5.1±0.9	5.2±0.9	5.3±0.9
TG, mmol/L	1.6 (1.1–2.1)	1.1 (0.8–1.5)**	1.4 (1.0–1.9)**	1.8 (1.3–2.7)**
HDL-c, mmol/L	1.2 (1.0–1.5)	1.4 (1.2–1.7)**	1.3 (1.1–1.5)**	1.2 (1.0–1.4)**
LDL-c, mmol/L	3.1±0.7	3.0±0.8	3.2±0.8	3.3±0.8
CRP, mg/L	0.93 (0.56–1.73)	0.64 (0.38–1.30)**	0.91 (0.55–1.75)**	1.39 (0.73–2.59)**
Cr, umoI/L	66 (56–76)	62 (54–74)*	63 (55–75)	60 (52–73)*
Hypertension, *n* (%)	84 (57.53)	274 (37.85)**	126 (56.00)	274 (60.35)
Diabetes, *n* (%)	26 (17.81)	90 (12.43)	49 (21.78)	140 (30.84)*
Dyslipidemia, *n* (%)	52 (35.61)	170 (23.48)*	72 (32.00)	216 (47.58)**
Use of antihypertensive agents, *n* (%)	43 (29.45)	119 (16.44)	76 (33.78)	164 (36.12)
Use of hypoglycemic agents, *n* (%)	14 (9.59)	40 (5.52)	23 (10.22)	56 (12.33)
Use of lipid-lowering agents, *n* (%)	7 (4.79)	18 (2.49)	9 (4.00)	28 (6.17)

Continuous variables are expressed as means±standard deviation or medians with interquartile range. Categorical variables are expressed as numbers with percentages.

* *p* < 0.05,

** *p* < 0.01. BMI, body mass index; NC, neck circumference; WC, waist circumference; SBP, systolic blood pressure; DBP, diastolic blood pressure; FPG, fasting plasma glucose; 2hPG, 2-h plasma glucose; FINS, fasting insulin; HbA_1c_, glycated hemoglobin A_1c_; HOMA-IR, homeostasis model assessment-insulin resistance index; Alb, albumin; ALT, alanine aminotransferase; AST, aspartate aminotransferase; GGT, γ-glutamyl transpeptidase; ALP, alkaline phosphatase; Cr, creatinine; TC, total cholesterol; TG, triglyceride; HDL-c, high-density lipoprotein cholesterol; LDL-c, low-density lipoprotein cholesterol; CRP, C-reactive protein.
